# Transcriptome analysis of egg viability in rainbow trout, *Oncorhynchus mykiss*

**DOI:** 10.1186/s12864-019-5690-5

**Published:** 2019-04-27

**Authors:** Hao Ma, Kyle Martin, Doug Dixon, Alvaro G. Hernandez, Gregory M. Weber

**Affiliations:** 1 USDA/ARS National Center for Cool and Cold Water Aquaculture, Kearneysville, WV USA; 2 Troutlodge Inc., Sumner, WA USA; 3 University of Illinois at Urbana-Champaign, Urbana, IL USA

**Keywords:** Rainbow trout, Egg quality, Polyadenylation, mRNA, Mitochondria

## Abstract

**Background:**

Maternal transcripts are accumulated in the oocyte during oogenesis to provide for protein synthesis from oocyte maturation through early embryonic development, when nuclear transcription is silenced. The maternal mRNAs have short poly(A) tails after undergoing post-transcriptional processing necessary for stabilizing them for storage. The transcripts undergo cytoplasmic polyadenylation when they are to be translated. Transcriptome analyses comparing total mRNA and elongated poly(A) mRNA content among eggs of different quality can provide insight into molecular mechanisms affecting egg developmental competence in rainbow trout. The present study used RNA-seq to compare transcriptomes of unfertilized eggs of rainbow trout females yielding different eyeing rates, following rRNA removal and poly(A) retention for construction of the libraries.

**Results:**

The percentage of embryos to reach the 32-cell stage at 24 h post fertilization was significantly correlated to family eyeing rate, indicating that inviable embryos were developmentally compromised before zygotic genome activation. RNA sequencing identified 2 differentially expressed transcripts (DETs) from total mRNA sequencing comparing females with low-quality (< 5% eyeing), medium-quality (30–50% eyeing), and high-quality (> 80% eyeing) eggs. In contrast, RNA sequencing from poly(A) captured transcripts identified 945 DETs between low- and high-quality eggs, 1012 between low- and medium-quality eggs, and only 2 between medium- and high-quality eggs. The transcripts of mitochondrial genes were enriched with polyadenylated transcript sequencing and they were significantly reduced in low-quality eggs. Similarly, mitochondrial DNA was reduced in low-quality eggs compared with medium- and high-quality eggs. The functional gene analysis classified the 945 DETs between low- and high-quality eggs into 31 functional modules, many of which were related to ribosomal and mitochondrial functions. Other modules involved transcription, translation, cell division, apoptosis, and immune responses.

**Conclusions:**

Our results indicate that differences in egg quality may be derived from differences in maternal nuclear transcript activation and cytoplasmic polyadenylation before ovulation, as opposed to accumulation and storage of maternal nuclear transcripts during oogenesis. Transcriptome comparisons suggest low-quality eggs suffered from impaired oxidative phosphorylation and translation. The DETs identified in this study provide insight into developmental competence in rainbow trout eggs.

**Electronic supplementary material:**

The online version of this article (10.1186/s12864-019-5690-5) contains supplementary material, which is available to authorized users.

## Background

Reliable production of high-quality eggs is essential for meeting production cycle demands for seed stock. Fertility is high in the rainbow trout industry when fish are maintained under optimal conditions. Nevertheless, quality of the eggs or ova can be affected by many intrinsic and extrinsic factors including the genetics, age and diet of brood fish [1–6]; pre-spawning exposure to stressors and photo-thermal cycles [7–10]; and postovulatory aging of the eggs [11–13]. Understanding mechanisms by which egg quality becomes compromised in response to suboptimal genetics, management, nutrition, and environmental conditions is critical to optimizing hatchery productivity.

The oocyte becomes transcriptionally inactive following oocyte growth and remains transcriptionally silent or greatly repressed until zygotic genome activation (ZGA) which usually takes place around the time of the mid-blastula transition (MBT) in most vertebrates. Therefore, the oocyte serves as a reservoir of biomolecules including proteins, lipids and RNAs deposited into the egg during oogenesis, for utilization from oocyte maturation through early embryonic development [14, 15]. Levels of certain proteins and lipids have been linked to egg viability in many fish species including rainbow trout [16]. A relationship between the maternal transcriptome and developmental competence has also been supported in a variety of fishes using an assortment of molecular approaches, although few investigations involved next-generation sequencing [17, 18]. In rainbow trout, genes linked to decreased egg quality caused by postovulatory aging were identified using quantitative reverse transcription PCR [19–21] and transcripts associated with decreased egg quality in response to the use of photoperiod to shorten the time to spawning or hormone-induced ovulation were identified by microarray analyses [22, 23]. One microarray study compared the transcriptome of un-manipulated female rainbow trout that exhibited either 100% viability through eyeing or less than 64%, but confirmed only one differentially expressed transcript (DET) [24]. Less than 200 DETs were identified among the studies with few overlapping transcripts between them. Although deep sequencing approaches have been used to identify some miRNAs and mitochondrial genome encoded small RNAs related to egg deterioration due to postovulatory aging [25, 26], global mRNA analysis techniques based on deep-sequencing technologies have not been applied to investigate a possible connection between mRNA content in unfertilized eggs and egg quality in fish.

Stored maternal transcripts generally have shortened polyadenylic acid (poly(A)) tails and are masked to inhibit both translation and degradation [27–30]. In the oocyte, new transcripts intended for sequestration are polyadenylated in the nucleus and then translocated to the cytoplasm where they are subsequently partially deadenylated for storage [30–32]. The stored maternal transcripts require cytoplasmic polyadenylation to allow translation. The cytoplasmic polyadenylation or deadenylation of stored transcripts is a critical control mechanism for translation during oocyte maturation and early embryonic development [28, 33, 34]. When comparing eggs of different quality, differences in expressed transcripts based on total mRNA content may indicate differences in accumulation or degradation of the transcripts throughout oogenesis and maturation, whereas differences in mRNAs with longer poly(A) tails may indicate differences and changes in the translational activity of the transcripts [35–38]. To further understand the relationship between the maternal transcriptome and egg quality, we used RNA-seq to compare mRNA transcriptomes of ovulated eggs from 20 individual females that produced eggs of disparate quality as determined by eyeing rate. We compared transcriptomes sequenced from libraries prepared following rRNA removal or by oligo(dT) capture of polyadenylated RNA. Oligo(dT) capture methodologies are not efficient at capturing transcripts with short poly(A) tails [35, 39, 40], and therefore, mRNA transcriptomes sequenced from libraries prepared by oligo(dT) capture of polyadenylated RNA should be enriched in activated transcripts with elongated poly(A) tails.

## Results and discussion

### Eyeing rate and early embryo viability

**Fig. 1:**
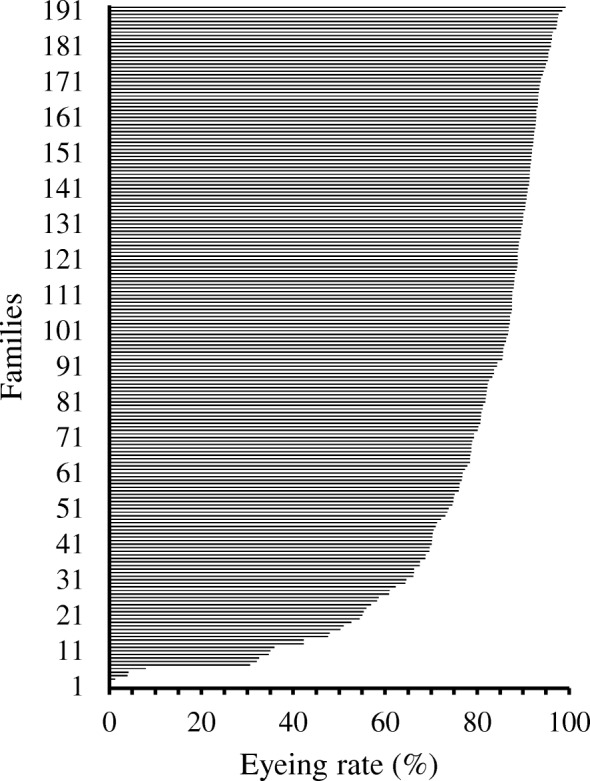
Eyeing rates of the 192 surveyed rainbow trout families in the selective breeding program

Viability was assessed at ~ 250 accumulated thermal units (ATUs) post fertilization, which we refer to as eyeing in the present manuscript. The 250 ATU mark is actually after retinal pigmentation but is often used by industry because mortality is generally very low after retinal pigmentation [41]; it is well after embryos are resistant to mechanical shock [42, 43], and is several days before hatching which allows time for the eggs to be sorted to remove dead and subviable eggs before shipment to production facilities. Eyeing rates for the families in the selective breeding program was typical of rainbow trout aquaculture operations [5], exhibiting a mean eyeing rate of 79.3% (Fig. 1). Only 16 of the 192 families evaluated had less than 50% eyeing and were classified as subfertile. Furthermore, only six families exhibited an eyeing rate of less than 10%, whereas eyeing rates were greater than 30% for all others. Sperm used to fertilize each of the subfertile families also yielded families with eyeing rates over 78%, substantiating the eggs and not the sperm as the cause of the subfertility. Visual inspection of eggs collected before fertilization did not show obvious signs of the eggs being compromised in ways that would allow for their being discarded by hatchery personnel.

Embryo cleavage was assessed for the 20 selected families at about 24 h post fertilization (Table 1). On average about 80% of the embryos in the high-quality families reached at least the 32-cell stage at this time. The percentage of embryos reaching the 32-cell stage was similar to the eyeing rates for most families, suggesting that most of the embryos that would not survive had died or had very delayed development by the 32-cell stage. The percentage of embryos reaching the 32-cell stage was significantly correlated to eyeing rate (R = 0.85; *P* < 0.001). Few embryos failed to reach the 8-cell stage by 24 h post fertilization. Together these data support most of the non-viable embryos were fertilized but failed developmentally before the 32-cell stage, which should be before the major wave of ZGA. Although the timing of the major wave of ZGA has not been characterized in rainbow trout, in general, more cleavage divisions are completed before ZGA in animals that develop more slowly [44]. The ZGA for most fish species investigated, all of which develop more rapidly than rainbow trout, have been shown to begin during the MBT at about cell cycle 10 or ~ 1000 cells [45–49]. An exception is the medaka (*Oryzias latipes*) in which the ZGA begins at about the 64-cell stage; before the MBT [50]. A previous study on the same rainbow trout broodstock population as in the present study reported most embryo mortality in subfertile families took place by the second cleavage interval [51]; earlier than in our study and well before ZGA. Although the timing of embryonic mortality cannot specify a cause of the mortality, most of the embryonic mortality reported for this population of rainbow trout has taken place while the embryos were dependent upon maternal transcripts and before many of the other forms of stored biomolecules would be either required or exhausted by the embryos, leaving aberrations in levels or activation of maternal transcripts as possible contributors to subfertility.

**Table 1:** Assessment of early embryo development in 20 selected families. The percentage of embryos reaching each cell stage by ~ 24 h post fertilization, and eyeing rate, are indicated

Egg quality group		Family ID #	Embryos collected at ~ 24 h post fertilization						Eyeing rate (%)
			Total embryos	Embryos with ≥2 cells (%)	Embryos with ≥4 cells (%)	Embryos with ≥8 cells (%)	Embryos with ≥16 cells (%)	Embryos with ≥32 cells (%)	
Subfertile	Low quality	48	26	62	62	58	42	12	0.0
		63	42	81	81	74	21	0	1.3
		129	31	97	97	90	42	0	0.0
		136	53	98	98	92	81	9	4.2
	Medium quality	13	30	93	93	93	93	63	30.6
		60	61	82	82	75	41	3	42.2
		71	54	91	91	91	69	13	47.5
		106	45	93	93	91	91	40	34.7
		108	43	88	88	88	53	19	35.9
		114	49	100	100	100	94	65	42.2
Fertile	High quality	19	32	94	94	94	94	63	87.6
		47	25	100	100	100	100	88	83.6
		58	39	92	92	92	92	59	80.6
		59	52	98	98	98	96	83	97.7
		87	50	98	98	98	98	84	89.1
		97	54	94	94	94	94	81	87.4
		99	51	100	100	100	100	100	92.6
		102	59	100	100	100	100	95	92.1
		103	34	100	100	100	100	100	97.5
		119	47	98	98	98	96	87	88.8

### Mapping of sequencing reads following rRNA removal and poly(a) retention

RNA-seq analysis of the eggs of 20 females generated 31 to 58, and 43 to 68 million reads from the libraries constructed with the Illumina® TruSeq® Stranded Total RNA Library Prep Kit with Ribo-Zero Gold (rRNA removal) and TruSeq® Stranded mRNA Sample Prep Kit (poly(A) retention) respectively (Table 2). Similar percentages of the reads were mapped to rRNA gene sequences using the two approaches, with an average of 5.4% derived from the libraries constructed by rRNA removal and 4.7% derived from the libraries constructed by poly(A) retention kits respectively. Whereas the percentage of reads mapped to the nuclear transcriptome was half as great with poly(A) retention compared with rRNA removal, the percentage of reads mapped to mitochondrial RNA was more than 10-fold greater for poly(A) retention compared with rRNA removal. This reversal in trends is consistent with a higher proportion of the mitochondrial mRNA transcripts being polyadenylated than the proportion of the nuclear mRNA transcripts that are polyadenylated or efficiently captured by the poly(A) retention procedures.

**Table 2:** Overview of RNA-seq read alignments

Egg quality group	Female ID#	rRNA removal				Poly(A) retention			
		Total reads	Mapped to transcriptome (%)	Mapped to mitochondrial RNA (%)	Mapped to rRNA (%)	Total reads	Mapped to transcriptome (%)	Mapped to mitochondrial RNA (%)	Mapped to rRNA (%)
Low quality	48	37,157,250	43.6	3.1	8.3	61,999,239	30.3	32.7	2.4
	63	39,461,498	48.5	2.4	5.4	64,750,933	39.3	22.3	2.0
	129	58,281,853	54.8	2.4	3.7	59,599,504	39.1	18.4	3.3
	136	36,865,244	47.3	1.6	5.7	43,578,497	34.8	20.8	3.0
	Mean	42,941,461	48.6	2.4	5.8	57,482,043	35.9	23.6	2.7
Medium quality	13	37,890,847	44.9	3.0	7.6	64,816,319	20.9	53.9	5.7
	60	36,566,538	49.0	2.9	4.4	60,757,076	13.8	62.5	4.2
	71	33,120,194	51.2	5.0	4.1	55,561,085	12.9	61.2	12.7
	106	37,362,260	49.0	2.7	5.1	62,699,350	24.7	45.5	3.0
	108	53,796,039	52.9	6.8	5.5	61,835,604	24.5	45.6	2.6
	114	32,683,461	41.3	6.5	7.7	59,163,406	11.7	64.7	2.4
	Mean	38,569,890	48.1	4.5	5.7	60,805,473	18.1	55.6	5.1
High quality	19	37,448,462	46.1	3.3	7.7	66,890,967	32.3	35.5	2.8
	47	37,299,964	48.1	3.0	4.6	61,202,732	34.6	27.9	2.2
	58	36,637,099	43.5	2.1	6.2	61,065,293	37.6	26.6	1.8
	59	31,040,335	50.2	4.9	5.2	52,972,699	10.5	64.2	15.0
	87	38,856,780	49.7	2.8	5.0	55,700,910	18.8	53.3	5.9
	97	38,254,940	51.0	2.5	4.1	61,667,813	20.8	48.1	7.4
	99	55,069,888	52.7	4.8	3.2	68,472,008	25.4	46.3	1.7
	102	32,044,365	49.0	5.8	5.4	57,053,445	10.6	65.2	9.1
	103	39,749,239	48.1	1.7	4.1	59,550,700	23.8	43.2	4.3
	119	51,515,745	51.4	8.1	5.6	62,330,132	15.9	61.7	2.7
	Mean	39,791,682	49.0	3.9	5.1	60,690,670	23.0	47.2	5.3
All	Mean	40,055,100	48.6	3.8	5.4	60,083,386	24.1	45.0	4.7

Mitochondrial mRNA transcripts are polyadenylated with a tail of approximately 50 nucleotides in vertebrates as part of transcript processing and therefore most would be expected to be captured by the poly(A) retention [52, 53]. Similarly, nuclear mRNA transcripts are also polyadenylated as part of processing. In most cells the majority of cytosolic nuclear transcripts are polyadenylated with a poly(A) tail greater than 80 nucleotides [29, 54]. However, stored maternal nuclear transcripts possess a short poly(A) tail around 15–40 nucleotides that are elongated to over 80 nucleotides through cytoplasmic polyadenylation during activation [27, 29, 36, 40, 55]. In general, oligo(dT) capture approaches are not very efficient at capturing mRNAs with shorter poly(A) tails [35, 39, 40]. As far as we are aware, the capture efficiency of the Illumina® TruSeq® Stranded mRNA Sample Preparation Kit for short poly(A) tails such as those in stored maternal mRNA, has not been characterized. It is therefore likely that data collected following poly(A) retention represent primarily activated mRNAs with longer poly(A) tails and data following rRNA removal represent primarily the more abundant stored maternal transcripts with short poly(A) tails, which is consistent with a lower percentage of the transcript reads aligning to the nuclear transcriptome following poly(A) retention compared with rRNA removal.

**Fig. 2:**
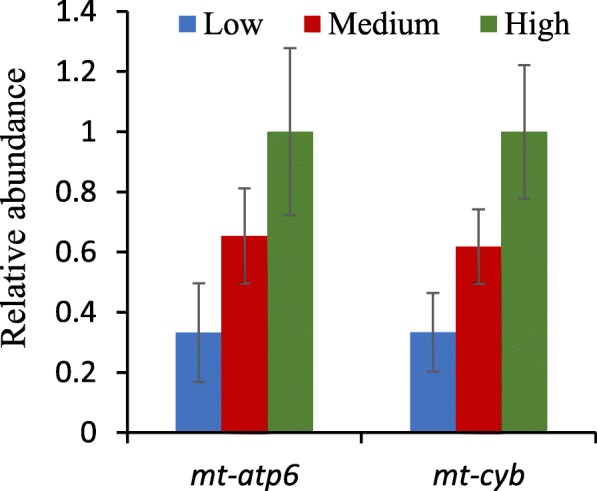
Relative abundance of mitochondrial DNA. Real-time quantitative PCR measurement of *mt-atp6* and *mt-cyb* genes normalized to 18S, in low-, medium-, and high-quality eggs. (mean ± SEM)

Eggs of vertebrates possess large stockpiles of mitochondria that are active in providing energy during maturation and stockpiling ATP for energy to drive early embryonic events including cleavage [56–59]. Furthermore, there is extensive mitochondrial DNA (mtDNA) replication during maturation that then ceases until after ZGA. High proportions of mitochondrial transcripts as observed in our study have been reported in early cod and halibut embryos [48, 60]. The percentage of reads that were aligned as mitochondrial transcripts was reduced in the low-quality eggs following poly(A) retention as well as rRNA removal. The percentage of reads aligned to mitochondrial RNAs in the low-quality eggs following poly(A) retention was 23.6% compared with 47.2% in the high-quality eggs, and 2.4% compared with 3.9% respectively following rRNA removal (Table 2). A reduction in mitochondria and mtDNA has been identified as a cause of reduced fertility in several mammalian species and serves as a marker of oocyte quality in mammals [56, 57]. Furthermore, augmentation of mitochondrial number or mtDNA in maturing oocytes has been recognized as a method to improve oocyte quality in mammals including humans [56, 57, 61, 62]. We therefore used real-time quantitative PCR to measure abundance of mtDNA in our samples by measuring two mitochondrial genes, *mt-atp6* and *mt-cyb*. Abundance of both genes were reduced 1.6 log2 fold change (log2FC) in the low-quality eggs (Fig. 2) suggesting a reduction in mitochondria or mtDNA may contribute to differences in the percentage of reads aligned to the mitochondrial transcriptome among egg quality groups.

### Gene expression profiling revealed greater differences in polyadenylated mRNA than total mRNA abundance with fertility

**Fig. 3:**
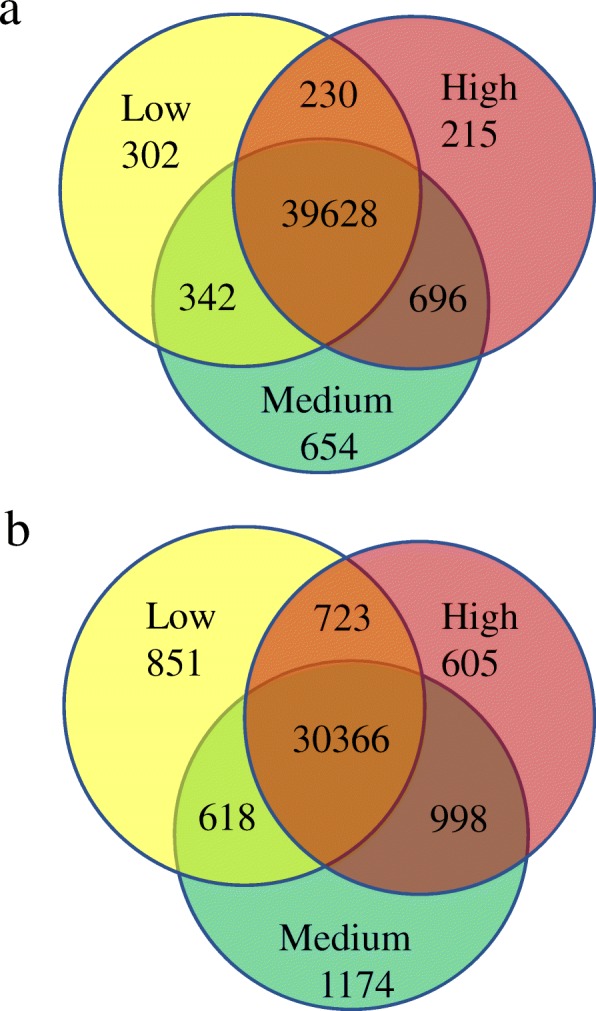
Gene transcripts detected in low-, medium-, and high-quality eggs. **a** Libraries constructed by rRNA removal. **b** Libraries constructed by poly(A) retention. Genes expressed with average normalized reads greater than 1 were counted

We detected 44,330 and 39,133 transcripts with at least three normalized reads expressed in the libraries constructed by rRNA removal and poly(A) retention kits respectively. About 89% of the transcripts following rRNA removal and 77% of the transcripts following poly(A) retention were shared among the three treatment groups (Fig. 3) and many of those that were not shared had low transcript numbers. No DETs were identified comparing the 10 subfertile and 10 fertile or high-quality females from either the rRNA removal or poly(A) retention libraries by DESeq2 with the criteria of a false discovery rate (FDR) < 0.05. We therefore divided the subfertile group into a low- and medium-quality group (Table 1).

Only two DETs were identified among the three treatment groups; low-, medium-, and high-quality eggs, from our dataset sequenced from the libraries constructed with an rRNA removal kit by DESeq2 with the criteria of log2FC ≥ 1, FDR ≤ 0.05, and a total number of normalized reads in a given comparison being ≥500 (Additional file 1: Table S1). The transcript *agfg1* (Arf-GAP domain and FG repeats-containing protein 1) was enriched in low-quality eggs compared with high-quality eggs (1.81 log2FC, FDR = 0.0172) and *ahnak* (neuroblast differentiation-associated protein ahnak-like) was enriched in low-quality eggs compared with medium-quality eggs (2.01 log2FC, FDR = 0.0026). AGFG1 has been shown to affect the accumulation of a small set of non-polyadenylated cellular mRNAs in mammalian cells [63], and mRNA of Drongo (Drosophila neural GTS1-like), the homolog of AGFG1 in the fruit fly, associates with Me31B (maternal expression of 31B) which is required for the translational repression of maternal mRNAs in the oocyte [64]. AHNAK is considered to be involved in calcium flux regulation and has been proposed to interact with s100 proteins to regulate cellular Ca^2+^ homeostasis [65]. Notably, an s100 protein, *s100a1* (S100 calcium binding protein A1), is among the most reduced polyadenylated transcripts in low-quality eggs. Most important, the lack of DETs following rRNA removal supports the concept that low fertility was not a result of differences in the accumulation or degradation of transcripts throughout oogenesis or maturation.

A total of 1339 transcripts were differentially expressed among the egg quality groups when the libraries were constructed with a poly(A) retention kit, using the same criteria as with rRNA removal (Additional file 1: Table S1). There were only 2 DETs comparing medium- and high-quality eggs, *esr2b* (estrogen receptor beta 2) and *pltp* (phospholipid transfer protein), both of which were higher in medium quality eggs compared with both low- and high-quality eggs. There were 945 DETs comparing low- and high-quality eggs, and 1012 comparing low- and medium-quality eggs. There were 619 DETs shared between the low- versus high-quality egg comparison and the low- versus medium-quality comparison, and both DETs from the medium- versus high-quality comparison were also differentially expressed between the low- and medium-quality eggs. The shared DETs between the low- versus high- quality comparison and the low- versus medium-quality comparisons were consistent in direction of change for any given transcript. Taken together, there was little difference in the transcriptomes of medium-quality eggs with between 30 and 50% eyeing rates, and the high-quality eggs with greater than 80% eyeing rates; therefore, we focused our further analyses and discussions on the low- versus high-quality comparison. The 945 DETs included 732 unique gene descriptions, with many of the more differentially expressed transcripts sharing gene descriptions with multiple transcripts or locus tags.

**Fig. 4:**
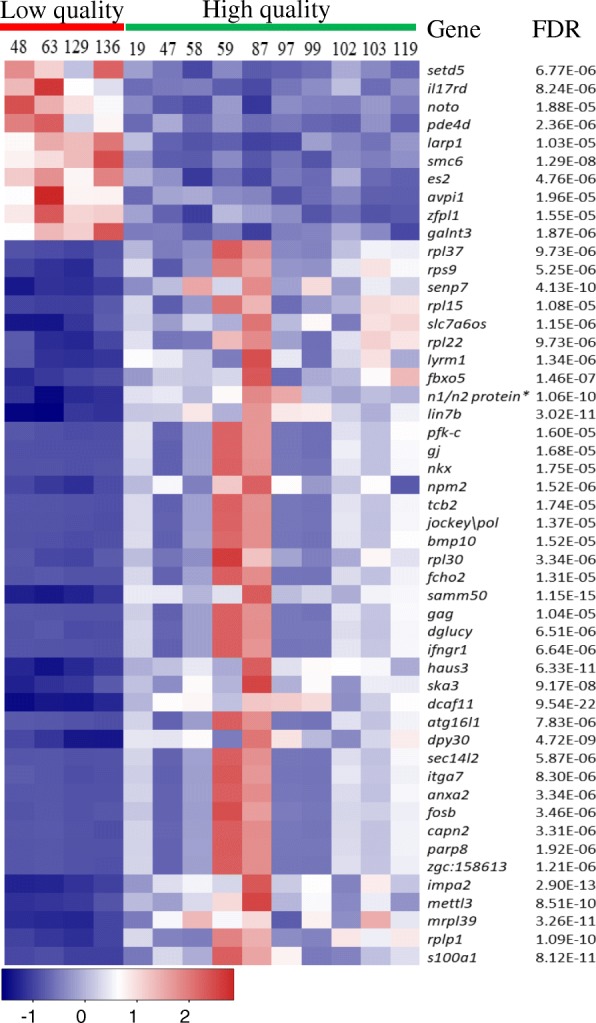
Heat map of 50 differentially expressed transcripts. The top 10 up-regulated and 40 down-regulated genes in low-quality eggs were selected based on false discovery rate (FDR) value. The red bar indicates females with low-quality eggs and the green bar indicates females with high-quality eggs. * Indicates the gene has not been officially named. The gene name abbreviations are listed in Additional file 1: Table S7

Among those 945 DETs, 724 were decreased and 221 were increased in low-quality eggs (Additional file 1: Table S1). There were many nuclear genes associated with ribosome production and function among the most significantly reduced DETs. Over 100 of the 945 DETs were for 40s or 60s ribosomal proteins. In general, the functions of the transcripts were diverse even among the most differentially expressed transcripts (Fig. 4). The transcripts in Fig. 4 were associated with 62 different GO terms for Biological process and 30 for Molecular function, even with 14 of these transcripts having no associated GO terms. Among the 221 enriched transcripts in the low-quality eggs, the gene transcript with the largest increase, 2.07 log2FC, is *tob1* (protein tob1-like) (Additional file 1: Table S1). This transcript, however, was highly variable among the low-quality groups (FDR = 0.0010). Whereas the high-quality eggs ranged from 46 to 280 normalized reads, one of the low-quality groups with no survival at eyeing and another with 1.3%, had over 1300 normalized reads each, whereas the other two had below 80 reads. TOB1 is characterized as an anti-proliferative protein whose activity is mediated through interactions with the Caf1a/Caf1b deadenylases leading to target mRNA deadenylation and decay [66–68]. Considerable differences in expression among the groups of low-quality eggs, including a transcript involved with mRNA deadenylation and decay, might suggest disparate causes leading to the reduction in quality or differences in the progression of viability among the low-quality families.

Because egg quality is a major issue in the aquaculture industry [69], many microarray and quantitative reverse transcription PCR studies have revealed sets of genes associated with egg quality [17]. In rainbow trout, eggs with reduced egg quality in response to postovulatory aging had lower expression levels of *tubb* (tubulin b) and *npm2* (nucleoplasmin-2) [20], and eggs with reduced quality in response to photoperiod treatment to accelerate the time of spawning had increased expression of *pyc* (pyruvate carboxylase) [22]. Consistent with those findings, our data show transcript levels of *tubb* and *npm2* are decreased, and pyc is increased, in low-quality eggs. On the other hand, we observed decreased expression of *krt8* (keratin 8), *krt18* (keratin 18), *rpl24* (60s ribosomal protein l24), and *apoc1* (apolipoprotein c1) with reduced egg quality, whereas *krt8* and *krt18* have been reported to increase with reduced egg quality due to postovulatory aging [20], and *rpl24* and *apoc1* to increase with reduced egg quality in response to hormone implantation or photoperiod manipulation to accelerate the time of spawning [22]. The three studies used poly(A) retention and therefore likely measured changes in activated transcripts. In the present study total transcript abundance based on rRNA removal libraries did not significantly differ between high- and low-quality eggs for any of the seven genes mentioned, with the mean normalized reads differing by less than 10% for *rpl24*, *pyc*, *npm2*, and *tubb*, and 68, 80 and 146% for *krt8*, *krt18* and *apoc1* respectively. Moreover, the directions of the trends were similar with poly(A) retention and rRNA removal for each of these transcripts. The differences in the direction of the expression of specific genes among studies may be due to the treatments behind the reductions in egg quality or criteria for assessing developmental competence. Alternatively, differences may reflect where in the activation and deactivation cycle of the transcripts the eggs were sampled, and not just a measure of how many transcripts are activated overall. Further study of the kinetics of cytoplasmic adenylation and deadenylation in rainbow trout may help with interpretations of transcript levels and their associations with egg quality.

Even with poly(A) retention, only two transcripts were differentially expressed between medium- and high-quality eggs (*esr2b* and *pltp*) despite the medium-quality eggs ranging from 31 to 48% eyeing, and also representing only the lower 8% of clutches in terms of eyeing rate. The lack of DETs between medium- and high-quality eggs may suggest transcript variance did not contribute to the reduced egg quality, or transcript disparities were not detected due to the nature of the study or data analyses. Transcript signatures associated with less drastic differences in fertility have been identified in rainbow trout eggs in response to specific treatments [20, 22, 23]. Since the cause or causes of the reduced egg quality in the present study is undefined, there is likely a host of maladies among the egg clutches resulting in reduced fertility that do not share the same molecular pathways, making transcriptome signatures of any one difficult to discern. More robust analyses such as use of artificial neural networks and supervised machine learning which has been able to identify molecular signatures composed of many minor changes in transcript levels in oocytes of striped bass that associated with fertility [70] may be helpful. Regardless, the expressions of many genes have been linked to egg quality in the present study including genes not previously associated with egg quality, providing new insight into how an egg can become compromised.

### Mitochondrial RNA expression

The rainbow trout mitochondrial genome encodes 13 polypeptides, 22 tRNAs, two rRNAs and has a noncoding D-loop region [71]. Transcripts for the 13 mitochondrial protein coding genes and D-loop region were found to be significantly reduced in the low-quality eggs by RNA-seq with poly(A) retention libraries (Table 3). In addition, *mt-tn (tRNA-Asn)* was also decreased in low-quality eggs but few transcripts were detected for this or all other tRNA genes. There were also abundant reads for the rRNAs; *mt-rnr1* (mitochondrion 12S) and *mt-rnr2* (mitochondrion 16S), and despite the trends being similar for the rRNAs and mRNAs, the DESeq2 analysis did not detect a significant reduction in *mt-rnr1* or *mt-rnr2* in low-quality eggs (FDR > 0.1; Table 3). Although there are some reports of mitochondrial rRNAs being polyadenylated [72, 73], mitochondrial rRNAs are generally not polyadenylated or contain no more than 10 nucleotides of the tail in most vertebrate cells [52, 53, 74]. It is worth noting that whereas the rRNAs comprised about half of the mitochondrial RNA reads following rRNA removal (Additional file 1: Table S2), they comprised less than 5% of the mitochondrial RNA reads following poly(A) retention, supporting the idea that few of the rRNA transcripts were polyadenylated or possessed long poly(A) tails. We do not know if the antibodies used for rRNA removal recognize *mt-rnr1* or *mt-rnr2* to any extent. Mitochondrial rRNA transcripts are polyadenylated as part of the degradation process [72] and therefore the differences in polyadenylated rRNA transcripts among groups may represent differences in transcripts undergoing degradation.

**Table 3:** DESeq2 statistics for mitochondrial ribosomal RNA and differentially expressed mRNA transcripts

Gene	Base mean	Log2 fold change	P-value	FDR
*dlp*	119,812	−1.73	3.86E-05	1.94E-03
*mt-atp6*	1,338,170	−1.63	6.52E-05	2.89E-03
*mt-atp8*	113,992	−1.77	5.49E-05	2.58E-03
*mt-col*	8,076,707	−1.94	2.65E-05	1.46E-03
*mt-coll*	7,324,055	−1.88	2.09E-05	1.23E-03
*mt-colll*	4,570,645	−1.81	2.31E-05	1.31E-03
*mt-cyb*	8,530,201	−2.10	4.01E-06	3.51E-04
*mt-nd1*	914,918	−1.70	5.69E-05	2.63E-03
*mt-nd2*	418,048	−1.70	9.08E-05	3.76E-03
*mt-nd3*	265,015	−1.35	9.77E-04	2.05E-02
*mt-nd4*	994,427	−1.67	9.67E-05	3.96E-03
*mt-nd4l*	86,839	−1.73	8.50E-05	3.55E-03
*mt-nd5*	702,120	−1.55	3.57E-04	1.01E-02
*mt-nd6*	238,040	−1.64	1.14E-04	4.43E-03
*my-tn*	136	−1.80	5.90E-04	1.43E-02
*mt-rnr1*	327,067	−1.06	4.83E-02	2.43E-01
*mt-rnr2*	1,289,619	−1.30	1.47E-02	1.20E-01

The changes in expression of the 13 mitochondrial protein genes between high-quality and low-quality eggs are very consistent, ranging between − 1.34 and − 2.10 log2FC as calculated by DESeq2 (Table 3). This consistency may be due to coordinated regulation of the transcripts as they are all needed for oxidative phosphorylation [52, 57], or to a reduction in mitochondria in the low-quality eggs. However, DESeq2 uses shrinkage estimators [75] that resulted in reduced log2FC estimates for many DETs compared with calculating log2FC based only on normalized read values. If log2FC for the 13 protein mRNAs are calculated based simply on the normalized reads the average is about − 2.21 log2FC for the low-quality eggs compared with the high-quality eggs (Additional file 1: Table S2). This magnitude of change is greater than the − 1.6 log2FC difference in mitochondrial DNA (Fig. 2), supporting an additional reduction in expression beyond just a reduction in mitochondrial number although the measurements were conducted using different assays. It is also worth noting that using the same calculation approach, *mt-rnr1* and *mt-rnr2* are also about a 2.3 log2FC, suggesting similar circumstances behind the reduced levels of the rRNAs and the mRNAs in low-quality eggs.

Interestingly DESeq2 analysis of data from rRNA removal libraries revealed no significant differences in mitochondrial gene expression among egg quality groups (FDR > 0.999). Nevertheless, the numerical means for the protein and D-loop mRNAs, and rRNAs, were lower in the low-quality eggs compared to the high-quality eggs. Again, it is worth noting that whereas there was an average − 0.17 log2FC between high- and low-quality eggs for these transcripts with DESeq2 (Additional file 1: Table S1), the log2FC calculated based simply on the normalized reads averaged − 0.78 (Additional file 1: Table S2). This is in line with the differences in the proportion of aligned mitochondrial transcripts among treatments with rRNA removal libraries (Table 2). Differences in percentage of reads aligning with mitochondrial genes among groups are not consistent with a − 1.6 log2FC difference in mitochondrial DNA or mitochondrial numbers between low- and high-quality eggs. Among possible explanations for differences in mtDNA being greater than differences in total transcript reads for mitochondrial genes may lie in differences in the timing of transcription and mtDNA replication. During maturation there is an explosion in mtDNA replication, increasing 1000-fold in some species [59]. It is not known when the transcripts being measured in ovulated eggs are transcribed or processed in relation to this increase in mtDNA replication. Perhaps a lower content of mtDNA in low-quality eggs has not yet been reflected in transcription rates. Alternatively, there could be a compensatory increase in mitochondrial transcription in those oocytes with reduced mtDNA. The greater reduction in polyadenylated mitochondrial transcripts than mtDNA in low-quality eggs may reflect a combination of less mtDNA and transcript processing within mitochondria.

The requirement for oxidative phosphorylation by mitochondria to provide ATP in the early embryo varies among species [56, 76]. In the zebrafish, the maternal ATP pool is insufficient to execute the ubiquitin proteasomal pathway required for protein degradation required to advance beyond the 32-cell stage [76]. Although the 32-cell stage is well before ZGA in this species [45, 47], mitochondrial transcription has been shown to be active prior to mtDNA replication in zebrafish embryos [58]. Mitochondria in zebrafish embryos are active and free fatty acids serve as substrate for oxidative phosphorylation to supply the required ATP [76]. Less is known about how rainbow trout meet energy demands in the early embryo but oxidative metabolism is present even in the unfertilized eggs, and continues through early development [77]. Although the timing of the mortality in zebrafish embryos deficient in the ability to produce ATP is similar to that of the present study, egg ATP levels were found to not correlate with fertility in rainbow trout eggs [10, 77]. The associations among mitochondrial mtDNA abundance, mitochondrial transcript levels, ATP levels, and egg quality in rainbow trout are unresolved. Mitochondria serve many functions in addition to ATP production that are essential to embryo survival such as sequestration and release of intracellular calcium [78, 79]. Furthermore, deficiencies in mitochondrial activity and mtDNA number have been shown to have separate although overlapping impacts on egg quality [56, 80].

### Functional classification of differentially expressed genes

**Fig. 5:**
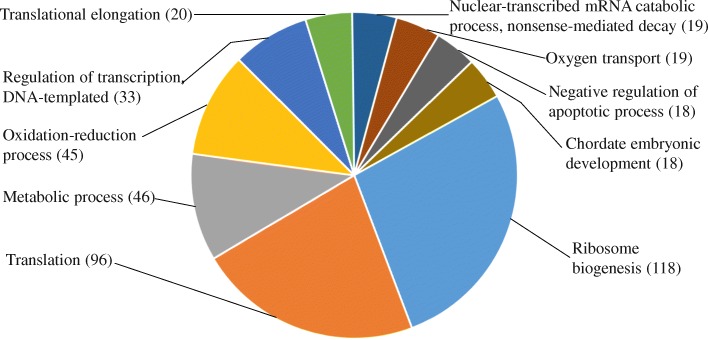
The 10 most represented gene ontology terms in biological process. The analysis included the 945 differentially expressed transcripts comparing low- and high-quality eggs. The number of significantly enriched gene ontology terms is shown in parenthesis, (*P* < 0.05)

Gene ontology (GO) analysis of the 945 DETs from the comparison of polyadenylated transcript enriched libraries for low- versus high-quality eggs revealed one or more associated GO terms for 811 of the transcripts. The five most common GO terms in Biological process are ribosome biogenesis, translation, metabolic process, oxidation-reduction process, and DNA templated regulation of transcription (Fig. 5). Clustering of DETs based on associated GO terms, using the Database for Annotation, Visualization and Integrated Discovery (DAVID) gene functional classification algorithms under kappa value of 0.3, resulted in 31 functional modules [81, 82] (Table 4). The enrichment scores of the modules ranged from 1.52 to 13.40 with the number of genes in each module ranging from 4 to 164, which included 547 of the DETs in total.

**Table 4:** Gene cluster analysis of differentially expressed genes

Cluster	Number of genes	Cluster enrichment	Maximum number of GOs in biological process		Maximum number of GOs in molecular function		Major involvement
			GO description	*P*-value	GO description	*P*-value	
Cluster 1	164	8.43	ribosome biogenesis	1.01E-130	structural constituent of ribosome	4.07E-159	Ribosome biogenesis
Cluster 2	74	5.82	transport	5.81E-03	transporter activity	2.56E-02	Mitochondrion related transporter
Cluster 3	55	2.09	autophagosome assembly	9.55E-04	protein binding	1.09E-05	Protein transport and modification
Cluster 4	48	2.44	proteolysis	9.53E-02	protein binding	1.09E-05	Protein transport
Cluster 5	32	3.22	metabolic process	4.74E-02	GTP binding	1.29E-04	Translation
Cluster 6	30	2.39	phosphorylation	4.84E-02	ATP binding	9.37E-03	Phosphorylation
Cluster 7	29	2.68	regulation of transcription, DNA-templated	7.14E-03	zinc ion binding	4.92E-02	Transcription
Cluster 8	22	13.4	rRNA processing	3.43E-07	nucleic acid binding	2.32E-02	Ribosome processing
Cluster 9	22	10.73	metabolic process	4.74E-02	metal ion binding	5.60E-02	Transcription
Cluster 10	20	10.7	oxygen transport	8.43E-20	iron ion binding	5.84E-07	Oxygen transport
Cluster 11	17	2.35	metabolic process	4.74E-02	zinc ion binding	4.92E-02	Zinc ion binding
Cluster 12	13	5.26	hydrogen ion transmembrane transport	6.72E-10	NADH dehydrogenase (ubiquinone) activity	5.13E-14	NADH dehydrogenase (ubiquinone) activity
Cluster 13	13	10.9	cellular iron ion homeostasis	1.69E-12	ferric iron binding	1.69E-16	Iron ion homeostasis and oxidation reduction
Cluster 14	12	2.96	ATP synthesis coupled proton transport	1.42E-11	proton-transporting ATP synthase activity	4.16E-09	ATP generation
Cluster 15	11	1.74	G-protein coupled receptor signaling pathway	1.22E-02	structural molecule activity	1.40E-02	structural molecule activity
Cluster 16	10	1.56	regulation of transcription from RNA polymerase II promoter	1.35E-01	nucleic acid binding	2.32E-02	Transcription
Cluster 17	10	5.65	oxidation-reduction process	2.83E-04	glutathione peroxidase activity	1.93E-06	Protect from oxidative damage
Cluster 18	9	1.7	signal transduction	7.68E-04	transposase activity	8.42E-07	Immune response,
Cluster 19	9	5.28	positive regulation of RNA polymerase II transcriptional preinitiation	4.86E-05	TBP-class protein binding	3.31E-04	Cell division
Cluster 20	8	1.86	regulation of cell proliferation	7.20E-02	calcium ion binding	6.47E-04	Regulate cell proliferation
Cluster 21	8	1.67	midbrain-hindbrain boundary structural organization	4.06E-04	transcription factor activity, sequence-specific DNA binding	1.32E-02	Transcription regulation
Cluster 22	7	4.46	multicellular organism development	2.96E-02	nucleic acid binding	2.32E-02	Cell cycle and division
Cluster 23	7	3.39	proton transport	1.63E-05	NADH dehydrogenase (ubiquinone) activity	5.13E-14	Mitochondrial electron transport
Cluster 24	6	1.76	interstrand cross-link repair	3.82E-03	metal ion binding	5.60E-02	Transcription regulation
Cluster 25	6	1.92	transmembrane transport	1.35E-02	ATP binding	9.37E-03	Regulate apoptosis
Cluster 26	6	5.01	heart contraction	1.13E-02	metal ion binding	5.60E-02	Transcription regulation
Cluster 27	6	1.52	microtubule-based movement	1.03E-01	metal ion binding	5.60E-02	Transcription regulation
Cluster 28	6	1.97	proteolysis	9.53E-02	zinc ion binding	4.92E-02	Regulate cell cycle and apoptosis
Cluster 29	5	1.7	metabolic process	4.74E-02	metal ion binding	5.60E-02	ATP binding
Cluster 30	4	2.96	regulation of transcription, DNA-templated	7.14E-03	DNA binding	5.37E-04	Transcription regulation
Cluster 31	4	10.23	neurotrophin TRK receptor signaling pathway	2.92E-07	structural constituent of ribosome	4.07E-159	Transcription regulation

The largest cluster included 164 transcripts involved in ribosome biogenesis of which only 7 were increased in low-quality eggs. As mentioned, over 100 DETs are for 40s and 60s ribosomal proteins. Moreover, the categories of the GO terms in gene clusters 2, 3, 4, 5, 7, 8, 9, and 31, are also associated with ribosome function. Clusters 2, 12, 14, 17, and 23 are involved in mitochondrial function. Together, many of the clusters are associated with translation and the production of energy by mitochondria to drive early cell division. The other gene clusters are mainly involved in regulation of transcription, cell division, apoptosis, and immune responses. Transcripts increased in low-quality eggs were distributed among 22 clusters.

There were 398 DETs that were not included in the clusters. This includes the 134 DETs without associated GO terms such as *n1/n2 protein* (histone-binding protein n1 n2-like), *senp7* (sentrin-specific protease 7-like) and *parp8* (kisutch poly [ADP-ribose] polymerase 8-like); and 264 that were orphan genes such as *mettl3* (n6-adenosine-methyltransferase 70 kda subunit), *bmp10* (bone morphogenetic protein 10-like), and *haus3* (haus augmin-like complex subunit 3-like). The listed transcripts are among the top DETs based on FDR values and therefore may also serve as important indicators of egg quality (see Fig. 4).

## Conclusions

The present study identifies differences in the transcriptome among ovulated eggs of different quality for which most of the mortality occurred between fertilization and the 32-cell stage, which is before ZGA. The identification of only two DETs by RNA-seq of libraries constructed by rRNA removal kits, compared with 1339 DETs derived from libraries following poly(A) retention kits, supports transcriptome differences with egg quality arose from differences in cytoplasmic polyadenylation or deadenylation of stored maternal transcripts as opposed to being the result of differences in the accumulation of maternal transcripts during oogenesis. Furthermore, few DETs were identified between medium- and high-quality eggs. Nine clusters of DETs which encompassed 375 DETs or about 40% of all DETs identified between high- and low-quality eggs, were associated with ribosome biogenesis and processing. The multitude of ribosome related DETs in low-quality eggs suggests inadequate ribosome production required for maternal mRNA translation, which could lead to a cascade of developmental dysfunction. This reduction in ribosomal gene expression was true of mitochondrial transcripts as well as nuclear transcripts. Moreover, mtDNA abundance was reduced by 1.6 log2FC in low-quality eggs compared with high-quality eggs further supporting the ability of the egg to provide energy following fertilization was compromised. In addition to genes associated with ribosome and mitochondrion biogenesis and function, GO analysis indicates levels of transcripts involved in the regulation of transcription, translation, cell division, apoptosis, and immune responses were altered in the low-quality eggs. Many of these genes have not previously been reported to contribute to egg quality in rainbow trout. The present study provides insights into how dysfunction of the egg transcriptome can affect developmental competence in fish eggs.

## Methods

### Sample collection

Eggs were collected from rainbow trout that were part of the selective breeding program at Troutlodge Inc. Sumner, WA, USA. Eggs from individual two-year-old broodstock rainbow trout were stripped into plastic bags. About 90 unfertilized eggs from each female were collected and immediately frozen in liquid nitrogen, and another 50 eggs were collected and placed into modified Davidson’s fixative [83] for examination to eliminate samples with overripe eggs or other abnormalities. The remaining eggs were fertilized with sperm harvested from neomales. The semen derived from each sire was used to fertilize eggs from two to three females. The fertilized eggs were incubated as individual families as part of the Troutlodge Inc. selective breeding program which evaluates eyeing rate at about 250 ATUs calculated as the sum of mean daily water temperature in degrees Celsius, which we refer to as eyeing in the present manuscript. The eggs were incubated at 10 °C for about the first 24 h post fertilization, after which time a sample of about 25–60 embryos were collected from each family and fixed in Stockard’s solution [84] to evaluate early embryonic survival and viability at about the 32-cell stage by enumerating the embryos reaching each stage of cell cleavage (Table 1). The frozen samples were kept in a − 80 °C freezer at Troutlodge Inc. until they were shipped on dry ice to the National Center for Cool and Cold Water Aquaculture (NCCCWA) after which they were again placed at − 80 °C until RNA isolation. The fixed samples were shipped at ambient temperature to NCCCWA for evaluation.

### Selection of rainbow trout females for RNA-seq analysis of eggs

Selection of egg samples for RNA-seq analysis was based primarily on eyeing rate. A range of 130–218 individuals from each family were examined for survival and viability at eyeing. Dead and subviable eggs included those that were unfertilized, had precipitated yolk in response to shocking the eggs, or were considered to have poorly developed eyes. We considered an eyeing rate of 50% as the demarcation between fertile and subfertile families. Only 16 of the 192 families generated had eyeing rates that were less than 50% (Fig. 1). In addition, the sperm from the sires used for each of these matings also yielded at least one family with an eyeing rate greater than 78% confirming the sperm used for fertilization was not the cause of the poor eyeing rates. Ten of the subfertile families (0–47.5% eyeing) and 10 fertile or high-quality families with eyeing rates greater than 80% (80.6–97.7% eyeing) that shared sires with the ten subfertile families, were selected for RNA-seq analyses. Since there were no families with eyeing rates between 10 and 30%, we subsequently further divided the subfertile families as low- (0–4.2%) and medium- (30.6–47.5%) quality families or females. Visual examination of the fixed eggs from these 20 females revealed no obvious signs of poor egg quality before fertilization.

### Assessment of early embryo development

All embryos collected at about 24 h post fertilization from each of the females selected for RNA-seq analysis were examined to determine viability. The fixed embryos were immersed in 0.5% methylene blue overnight. The cell number of each embryo was counted or confirmed to be greater than 32, using a stereo microscope (Nikon SMZ660). Those embryos with less than 32 cells were considered subviable.

### RNA isolation and sequencing

RNAs were isolated from frozen eggs which were homogenized in Tri Reagent (Sigma, St. Louis, MO) with a Qiagen Retsch MM300 TissueLyser Shaker Mixer Grinder Agitator Mill (Retsch Inc., Haan, Germany). Total RNA was isolated following the manufacturer’s protocol with the modification of using Phase Lock Gel (5 PRIME, Inc., Gaithersburg, MD, USA) and Phase Separation Reagent (Molecular Research Center, Cincinnati, OH, USA) to separate the aqueous phase from the organic phase. The isolated RNAs were further purified by lithium chloride precipitation and treated with DNase. The RNA integrity was evaluated by gel electrophoresis (Additional file 2: Figure S1), a NanoDrop ND-1000 (Thermo SCIENTIFIC, Wilmington, DE, USA) with λ260/280 great than 1.98, and a 2100 Bioanalyzer (Santa Clara, CA, USA) with RNA Integrity Number between 7.2 and 9.1 (Additional file 1: Table S3). DNaseI treated RNA was used to construct libraries with the Illumina® TruSeq® Stranded Total RNA Library Prep Kit with Ribo-Zero Gold (rRNA removal) and TruSeq® Stranded mRNA Sample Prep Kit (poly(A) retention). The libraries were sequenced by HiSeq2500 with 100 nt paired-end reads. Raw reads were deposited in NCBI Sequence Read Archive database (SRA accession: SRP108797). We sequenced the libraries for rRNA removal in two batches. The first batch of four samples (99, 119, 108, and 129) had less than 4% of the reads aligned to rRNA whereas the second batch of 16 samples contained 14.4 to 55.7% rRNAs indicating the rRNAs were not effectively removed (Additional file 1: Table S4). Hence, the 16 samples were re-sequenced using a newly purchased kit which resulted in the effective removal of rRNAs to below an average of 6% of the reads. The data from the three rRNA removal sequencing runs were included in subsequent transcriptome analyses.

### Data analysis

To classify reads as belonging to the nuclear transcriptome, the mitochondrial transcriptome, or as rRNA, after the adaptor was trimmed by bcl2fastq v2.17.1.14, the reads passing FastQC evaluation (http://www.bioinformatics.babraham.ac.uk/projects/fastqc), were respectively aligned to the nucleotide sequences corresponding to all CDS features annotated on the rainbow trout genome assembly (GCA_900005705.1) [85] with 72 additional genes selected from gene bank (Additional file 1: Table S5), the mitochondrial genome which includes 38 genes and the D-loop region [25, 86] and rRNA genes [87] by using Bowtie2 tool under default settings [88]. To avoid redundancy between the transcripts assigned to the mitochondrial and nuclear transcriptomes, we used the mitochondrial transcripts as queries to blast the rainbow trout [85] transcriptome data mentioned above. The blast results showed that GSONMT00007417001 was aligned with mitochondrial *mt-rnr2*, *mt-tl1*, and *mt-nd1,* and GSONMT00007419001 aligned with *mt-nd4l*. Therefore, GSONMT00007417001 and GSONMT00007419001 were removed from the nuclear transcriptome reference list. In addition, although *mt-rnr1* and *mt-rnr2* transcripts were included in the mitochondrial transcriptome, some of the rRNAs in the rRNA reference list have high similarity to these genes and therefore some of the reads aligned under rRNA may have been *mt-rnr1* and *mt-rnr2*. The raw reads aligned to nuclear transcriptome and mitochondrial mRNAs were merged and used as input to DESeq2 to identify DETs among groups at a FDR < 0.05, log2FC greater than 1, and total number of normalized reads within comparisons being greater than 500.

Gene ontology analysis of the identified DETs was conducted using Blast2GO PRO platform (BioBam Bioinformatics S.L., Spain) [89]. An R script written according to the algorithms proposed by the Database for Annotation, Visualization and Integrated Discovery (DAVID) gene functional classification was used to cluster the identified DETs into functional gene modules based on the results of the GO analyses [81, 82]. Module enrichment scores were generated by calculating the geometric mean of the *P*-values which were derived from hypergeometric test of the input gene sets, followed by negative log transformation of the geometric mean [90]. False discovery rate was calculated using the Benjamini-Hochberg procedure. Spearman correlation coefficient for eyeing rate and early embryo viability was estimated using R (R × 64 3.3.0).

### Mitochondrial gene quantification by real-time quantitative PCR

The insoluble materials leftover following homogenization in Tri Reagent during RNA isolation was mixed with 180 μl of 1 × TE buffer and shaken at speed setting 30 for 2 min with a Qiagen TissueLyser and then incubated for 10 min at room temperature. The mixture was centrifuged at 12,000 rpm for 10 min, and the supernatant was transferred to a tube containing Phase Lock Gel. The tube was shaken and centrifuged as above. The supernatant was collected, and DNA was isolated using a Quick-DNA™ Universal Kit (Zymo Research, Irvine, CA, USA). The DNA isolate was digested with RNaseA and the DNA was purified using a ZR-Duet™ DNA/RNA MiniPrep kit (Zymo Research, Irvine, CA, USA). The DNA from about 90 eggs was eluted in 20 μl of elution buffer and then diluted by adding 20 μl of water. The relative quantity of the *mt-atp6* and *mt-cyb* genes was measured on an ABI 7900HT sequence detection system (Applied Biosystems, Foster City, CA). Each reaction consisted of 1.5 μL of diluted DNA, 3 μl of each primer (5 μM) and 1× SYBR Green PCR Master Mix (Applied Biosystems, Foster City, CA). The thermal cycling profile was 50 °C for 2 min, 95 °C for 10 min and 40 cycles of 95 °C for 30 s, 60 °C for 20 s, and 72 °C for 30 s. A final dissociation step was performed to assess the specificity of the reaction. Relative quantification of the mitochondrial DNA was estimated by the standard curve method with three technical replications, and mean differences of the mitochondrial DNA were reported as relative change using the value for the high-quality eggs as a calibrator. An 18 s rRNA gene was used as the reference control [91]. Primer sequences are shown in Additional file 1: Table S6.

## Additional files

Additional file 1: **Table S1.** Differentially expressed transcripts among low-, medium- and high-quality eggs identified by DESeq2. **Table S2.** Mean and log2FC of normalized mitochondrial DET reads. **Table S3.** Quality analyses for RNAs treated with DNase and submitted for RNA sequencing. **Table S4.** Overview of RNA-seq read alignments from the libraries constructed with defective rRNA removal kit. **Table S5.** Additional gene transcripts used as reference. **Table S6.** Primers used in real-time quantitative PCR. **Table S7.** Gene name abbreviations. (XLSX 506 kb)

Additional file 2: **Figure S1.** Electrophoresis of egg RNAs isolated from different families selected for RNA sequencing. The families labeled in red are from the low-quality group; the families labeled in green are from the medium quality group; the families labeled in black are from the high-quality group. About 400 ng/sample of RNA was loaded to each well. Family 10 was not used for RNA sequencing. (PPTX 122 kb)

## Abbreviations

ATUs: Accumulated thermal units
DAVID: Database for Annotation, Visualization and Integrated Discovery
DET: Differentially expressed transcript
FDR: False discovery rate
GO: Gene ontology
Log2FC: Log2 fold change
MBT: Mid-blastula transition
mtDNA: Mitochondrial DNA
NCCCWA: National Center for Cool and Cold Water Aquaculture. Gene name abbreviations are presented in Additional file 1: Table S7
Poly(A): Polyadenylic acid
ZGA: Zygotic genome activation
