# Supplementary figures and images for: Transcriptome analysis of egg viability in rainbow trout, Oncorhynchus mykiss
Source: BMC Genomics. 2019 Apr 27;20:319. doi: 10.1186/s12864-019-5690-5 (PMC6486991; doi:10.1186/s12864-019-5690-5)

## Slide 1
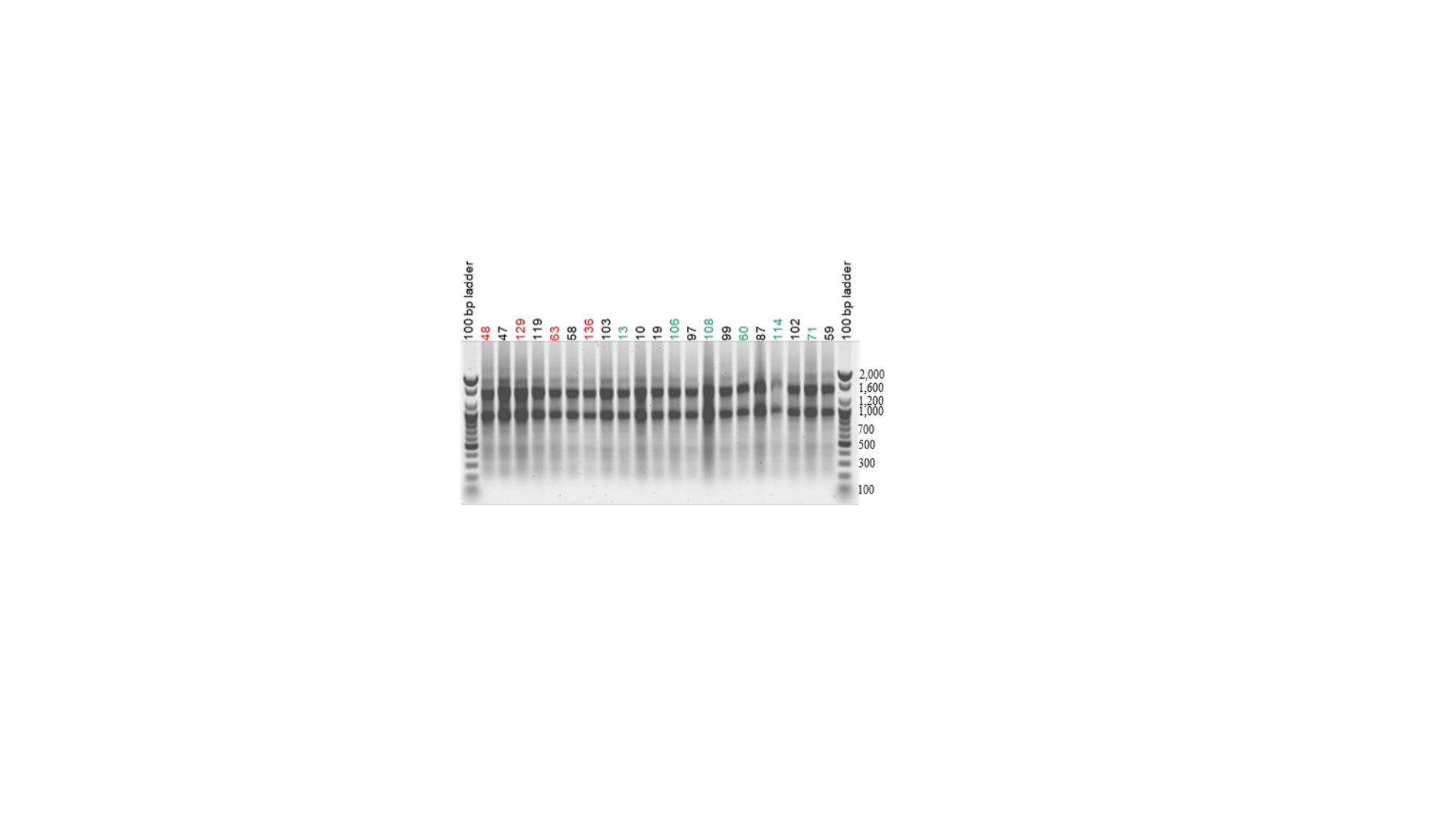

Supplement: Supplementary file 2 — Figure S1. Electrophoresis of egg RNAs isolated from different families selected for RNA sequencing. The families labeled in red are from the low-quality group; the families labeled in green are from the medium quality group; the families labeled in black are from the high-quality group. About 400 ng/sample of RNA was loaded to each well. Family 10 was not used for RNA sequencing. (PPTX 122 kb) [file 12864_2019_5690_MOESM2_ESM.pptx]
